# Reduced QSOX1 enhances radioresistance in nasopharyngeal carcinoma

**DOI:** 10.18632/oncotarget.23227

**Published:** 2017-12-14

**Authors:** Lei Zhou, Hong-Min Chen, Song Qu, Ling Li, Wei Zhao, Zhong-Guo Liang, Bin-Bin Yu, Kai-Hua Chen, Qi-Teng Lu, Guo-Xiang Lin, Xiao-Dong Zhu

**Affiliations:** ^1^ Department of Radiation Oncology, Affiliated Tumor Hospital of Guangxi Medical University and Cancer Institute of Guangxi Zhuang Autonomous Region, Nanning, Guangxi, P.R. China; ^2^ Guangxi Key Laboratory of Early Prevention and Treatment for Regional High Frequency Tumor, Guangxi Medical University, Nanning, Guangxi, P.R. China; ^3^ Key Laboratory of High-Incidence-Tumor Prevention and Treatment, Guangxi Medical University, Ministry of Education, Nanning, Guangxi, P.R. China; ^4^ Department of Oncology, Affiliated Wuming Hospital of Guangxi Medical University, Nanning, Guangxi, P.R. China

**Keywords:** QSOX1, nasopharyngeal carcinoma, radiosensitivity, CNE-2, radiotherapy

## Abstract

Radioresistance is a major cause leads to treatment failure in nasopharyngeal carcinoma (NPC). In our previous study, we identified that QSOX1 is a differentially expressed protein in NPC cell lines with variable radiosensitivities. The present study aimed to investigate the biological behavior of QSOX1 in nasopharyngeal carcinoma (NPC) and its effect on radiosensitivity. The levels of QSOX1 detected by enzyme-linked immunosorbent assay (ELISA) and immunohistochemistry (IHC) in radioresistant NPC patient sera and tissue samples were markedly lower than those in radiosensitive samples. Small hairpin RNAs (shRNAs) were employed to knock down endogenous QSOX1 expression in CNE-2 cells, and then, radiosensitivity, apoptosis, migration and invasion were assessed using colony formation, Cell Counting Kit-8 (CCK-8), flow cytometry, and transwell assays, respectively. Tumor growth and radioresistance were also evaluated using a xenograft model in nude mice. The shRNA-mediated knockdown of QSOX1 significantly increased cell survival under irradiation (IR) and weakened radiosensitivity, which was likely due to a reduction in the cell apoptosis rate after IR. Moreover, QSOX1 silencing led to the suppression of cellular migration and invasion. Similar results were obtained with the xenograft mouse model. Thus, targeting QSOX1 will provide a new avenue for increasing the sensitivity of NPC to radiotherapy.

## INTRODUCTION

Nasopharyngeal carcinoma (NPC) is a common head and neck malignancy that is prevalent in Southeast Asia, especially in southern China [1, 2]. Radiotherapy (RT) is the primary treatment option for this tumor type because of its predilection location and radiosensitivity [3]. Due to advances in radiological techniques, the survival of NPC patients has continued to improve. Nevertheless, local recurrence following RT remains a bottleneck that restricting the curative effect and favorable prognosis in NPC patients, and one of the main reasons behind this phenomenon is the radioresistance of NPC cells [4, 5]. It is well known that inter-individual variations in the genetic background of the tumor results in different therapeutic responses. Studies show that the occurrence of radioresistance may be related to radiation-induced gene regulation and expression [6, 7]. Therefore, the underlying mechanisms of NPC radioresistance need to be urgently explored to identify biomarkers for predicting NPC radiosensitivity and for guiding individualized treatment.

In our previous study, we analyzed the total secretory protein profiles in NPC cell lines with variable radiosensitivities and compared the conditioned serum-free medium of radioresistant CNE-2R cells with that of the parental radiosensitive CNE-2 cells using isobaric tags for relative and absolute quantitation (iTRAQ) with liquid chromatography-electrospray tandem mass spectrometry (LC-ESI-MS/MS) quantitative proteomics and found that the expression of quiescin sulfhydryl oxidase 1 (QSOX1) was significantly different between these cell lines [8]. Therefore, QSOX1 may be associated with the radiosensitivity of NPC.

The QSOX1 gene is located on chromosome 1q24 in humans, and it encodes two major isoforms by alternative mRNA splicing: QSOX1-S (66 kDa) and QSOX1-L (82 kDa) [9, 10]. The QSOX1 protein can be detected in the endoplasmic reticulum (ER), Golgi apparatus and secretory granules, as well as in the cell culture supernatant [11, 12]. QSOX1 belongs to the flavin adenine dinucleotide (FAD)-dependent sulfhydryl oxidase family that catalyzes the formation of disulfide bonds during protein folding [9, 13]. QSOX1 can reduce the oxidation of hydrogen peroxide and thus protect cell against oxidative stress [14, 15]. It is also involved in the remodeling of the extracellular matrix (ECM) [16]. Increasing evidence has demonstrated the association of QSOX1 with malignancy [17]. Recently, several studies have shown that the expression of QSOX1 is dysregulated in cancer cells and that QSOX1 is involved in tumorigenesis. Katchman BA *et al.* have reported that the overexpression of QSOX1 in pancreatic and breast tumors promotes cellular proliferation and invasion *in vitro*, that the expression of QSOX1 in breast tumors is correlated with tumor grade and that elevated QSOX1 mRNA in luminal B breast cancers is a predictive marker of poor prognosis [18, 19]. In another report, a correlation was observed between the overexpression of QSOX1 and the initiation of prostate tumor growth [20]. However, until now, the biological function and prognostic value of QSOX1 in head and neck squamous cell carcinoma, including NPC, have not been reported. In addition, the correlation between QSOX1 protein expression and radiosensitivity of NPC has not yet been determined.

In this study, we investigated the expression and biological behavior of QSOX1 in NPC and evaluated its effect on radiosensitivity. The results of IHC and ELISA assays showed that the levels of QSOX1 were down-regulated in radioresistant NPC samples. Then, by using the QSOX1 silencing cellular model and the xenograft mouse model, we demonstrated that reduced QSOX1 expression contributes to the radioresistance of NPC both *in vitro* and *in vivo*.

## RESULTS

### QSOX1 expression in CNE-2 and CNE-2R cells

**Figure 1:**
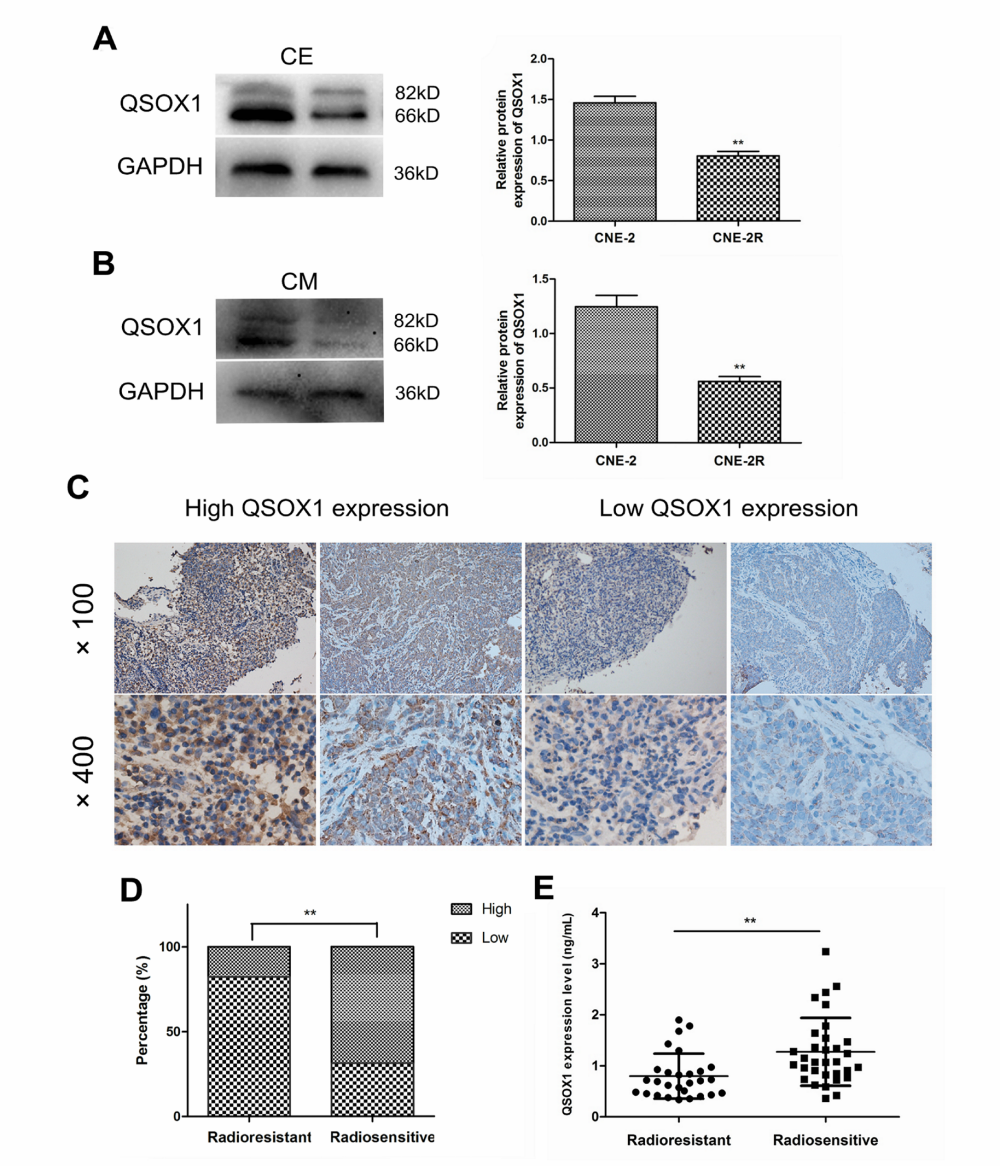
Expression of QSOX1 in NPC cell lines, serum and tumor tissues (**A** and **B)** Western blotting analysis of QSOX1 expression in cell extracts (CE) and conditioned media (CM) of CNE-2 and CNE-2R cell lines, and quantification of QSOX1 protein expression in cells. (**C)** Representative IHC images of QSOX1staining in tissue microarrays constructed from NPC patients with different radiosensitivities (original magnifications, 100× and 400×). **(D)** Expression rates of QSOX1 in the tissues of radioresistant and radiosensitive groups. (**E)** Serum concentration of QSOX1 in the two groups. ^*^p < 0.05 and ^**^p < 0.01.

To confirm differential expression of QSOX1 in NPC cell lines with variable radiosensitivities, western blotting was performed. As shown in Figure 1A, we first compared the QSOX1 levels in the cell extracts (CE) of CNE-2 and CNE-2R cells and found that QSOX1 was significantly down-regulated in the radioresistant CNE-2R cell line compared with the parental radiosensitive CNE-2 cells. Similarly, the QSOX1 levels were significantly different in the conditioned media (CM) of CNE-2 and CNE-2R cells (Figure 1B). Taken together, these results revealed that QSOX1 could be released into conditioned medium and may be related to radioresistance in NPC cells.

### Concentration of QSOX1 in NPC patients with different radiosensitivities

We determined the concentration of secreted QSOX1 in serum samples from 28 radioresistant and 32 radiosensitive NPC patients using enzyme-linked immunosorbent assay (ELISA). The level of QSOX1 was significantly different between the two groups (*p* = 0.001). As shown in Figure 1E, QSOX1 expression was low in the sera of patients with radioresistant NPC. TMA-based immunohistochemistry (IHC) measurements were performed to determine QSOX1 expression in NPC patients at the tissue level. QSOX1 expression was high in 17.9% (5/28) radioresistant NPC tissues, whereas it was 68.8% (22/32) in radiosensitive NPC tissues. (Figure 1D, *p* < 0.001). Positive staining was predominantly localized in the cytoplasm and extracellular matrix of NPC cells (Figure 1C). Taken together, these results were consistent with the results obtained using proteomics in our previous study.

### Successful stable knockdown of QSOX1 in CNE-2 cells

**Figure 2:**
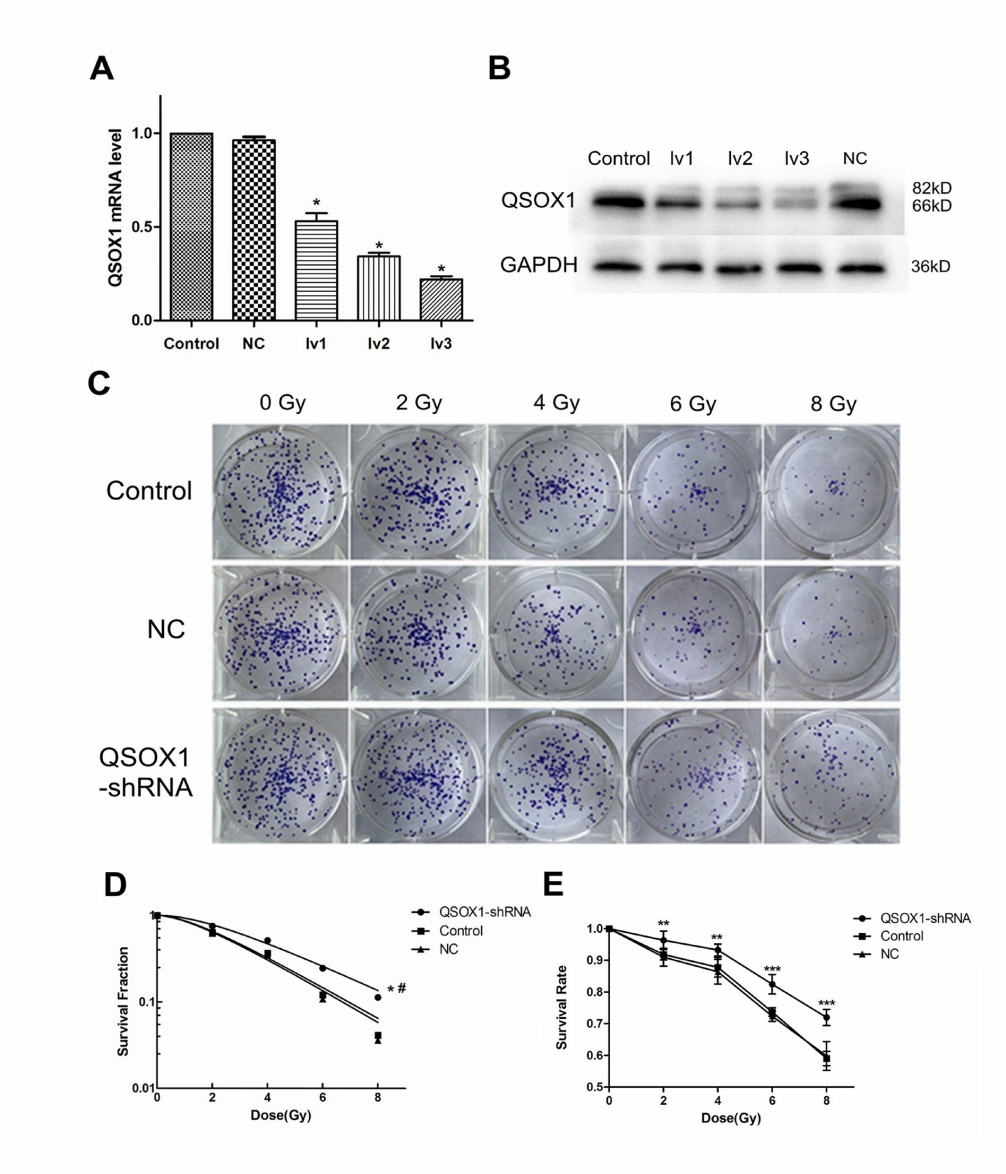
Knockdown of QSOX1 and its effect on radiosensitivity of NPC cells (**A)** RT-PCR analysis was performed to detect the expression of QSOX1 mRNA in different groups. (**B)** Western blot analysis demonstrating down-regulation of QSOX1 protein following shRNA transfection. (**C)** Representative colony formation images of control, NC and QSOX1-shRNA groups. (**D)** Dose-response curves were fitted according to the multi-target, single-hit model and analyzed using GraphPad Prism 5.0 software. ^*^p < 0.05 vs. control; ^#^*p* < 0.05 vs. NC. (**E)** Knockdown of QSOX1 increased the survival rate of CNE-2 cells after IR, with significant differences for all doses of radiation. ^*^p < 0.05, ^**^p < 0.01 and ^***^p < 0.001.

Both RT-PCR and western blotting confirmed the efficiency of lentivirus-mediated QSOX1 silencing (Figure 2). Finally, we chose QSOX1-shRNA-lv3 for follow-up experiments involving QSOX1 silencing.

### QSOX1-silenced cells display enhanced radioresistance

Cell Counting Kit-8 (CCK-8) and colony formation assays were conducted to evaluate the radiosensitivity of cells exposed to different doses of radiation. CCK-8 assay results showed that the cell survival rate of the QSOX1-shRNA group was significantly higher than that of the control group after irradiation (IR) (Figure 2E), confirming that QSOX1 silencing inhibited the anti-proliferative effect of irradiation. The radiation dose-clonogenic survival curves revealed that the survival fraction of cells was significantly higher in the QSOX1-shRNA group than in the control and NC groups (Figure 2D, both *p* < 0.05). Next, we used the widely accepted multi-target, single-hit model to compare the radiosensitivity of different cell lines. The survival fraction (SF) was calculated using the formula SF = 1–(1–e^-D/D0^)^N^. The main biological parameters associated with radiotherapy are shown in Table 1. The QSOX1-shRNA group had a higher D_0_ (mean lethal dose), Dq (quasi-threshold dose required for sublethal damage) and SF_2_ (survival fraction with 2 Gy radiation) than the control group. In other words, shRNA-mediated QSOX1 silencing led to an increase in the fraction of surviving cells after IR. To calculate the sensitization enhancement ratio (SER), we divided the D_0_ of the control group by the D_0_ of the QSOX1-shRNA group and obtained a value of SER_D0_ = 0.81 < 1, suggesting that the QSOX1-silenced cells were less sensitive to radiation than the control cells.

**Table 1:** Correlation parameters in the multi-target, single-hit model

Group	SF_2_	D_0_ (Gy)	Dq (Gy)
Control	0.637	2.383	1.507
NC	0.623	2.314	1.443
QSOX1-shRNA	0.754	2.936	2.249

D_0_ is the single radiation dose of radiation producing a 37% survival rate; SF2 is the survival fraction with 2 Gy radiation; Dq is the quasi threshold dose required for sublethal damage.

### Knockdown of QSOX1 inhibits the migration and invasion of CNE-2 cells *in vitro*

**Figure 3:**
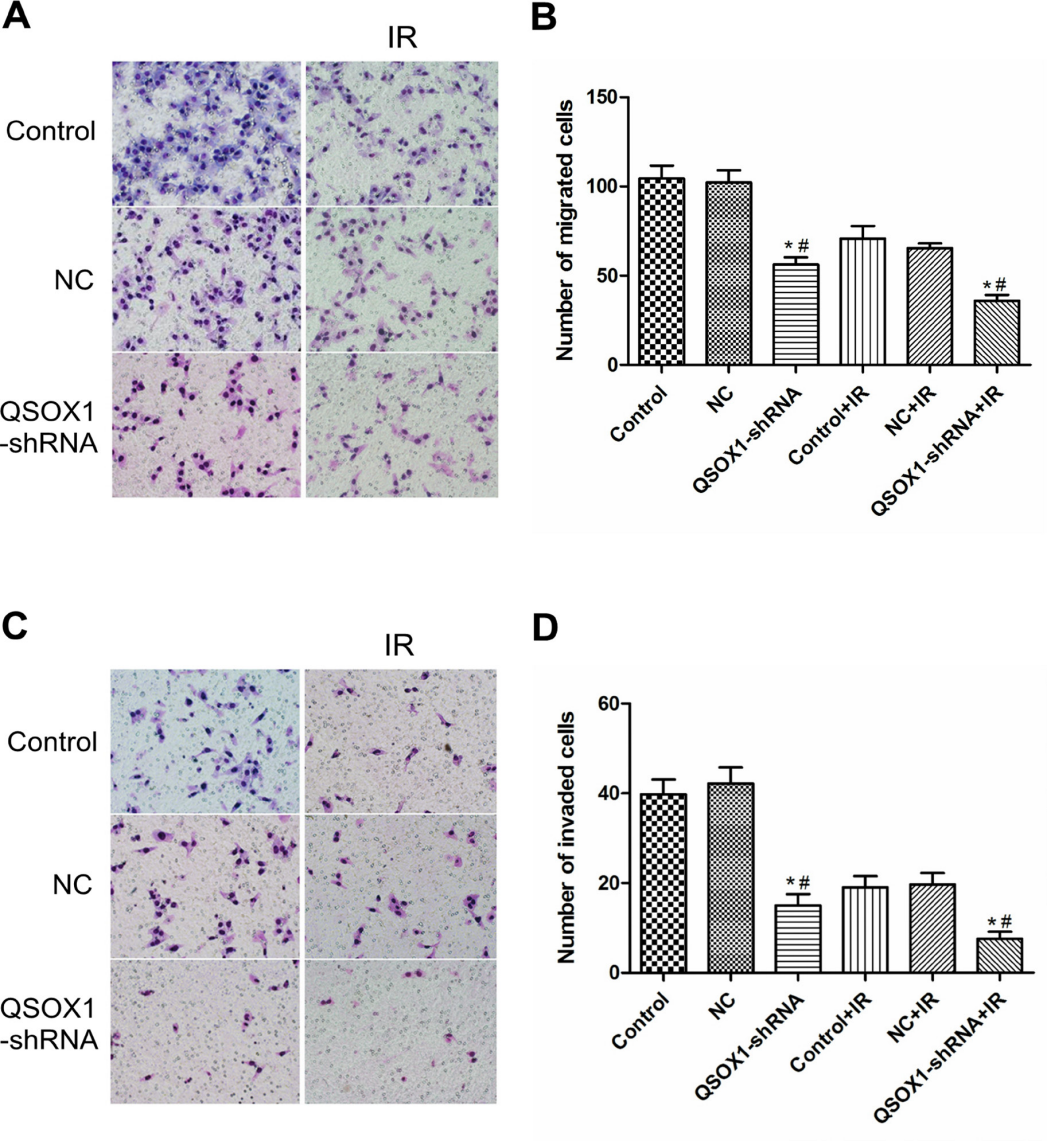
Effect of QSOX1 on cell migration and invasion **(A** and **C**) Representative micrographs depicting cell migration and invasion in the control, NC and QSOX1-shRNA groups (magnification, 200×). (**B)** Quantification of cells that had migrated through the transwell chambers. (**D)** Quantification of cells that had invaded through the transwell chambers precoated with Matrigel. ^*^p < 0.01 vs. Control; ^#^*p <* 0.01 vs. NC.

Migration and invasion through the basement membrane is a characteristic property of metastatic cancer cells. Using a transwell migration assay, we confirmed that the knockdown of QSOX1 significantly suppressed the migratory ability of CNE-2 cells both before and after 2 Gy IR (Figures 3A and 3B, *p <*0.01). Cell invasion was evaluated using a Matrigel-coated transwell chamber. Similarly, the invasive ability of the QSOX1-silenced cells was lower than that of the control and NC cells both before and after 2 Gy IR (Figures 3C and 3D, *p* < 0.01). Therefore, suppression of QSOX1 resulted in the loss of migratory and invasive abilities of NPC cells both in the absence and presence of irradiation.

### QSOX1-silencing results in apoptosis inhibition in CNE-2 cells after IR

**Figure 4:**
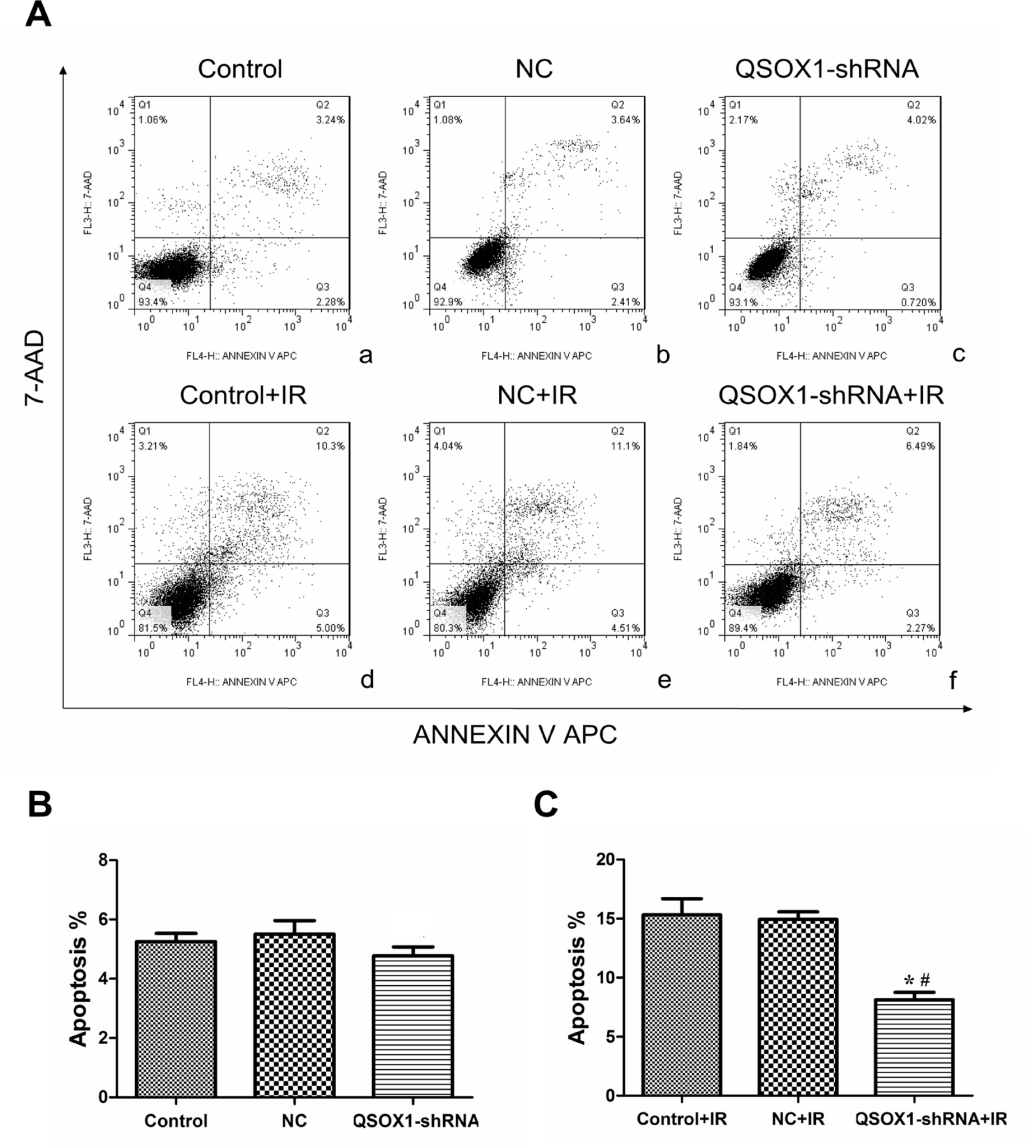
shRNA-mediated knockdown of QSOX1 decreased CNE-2 cell apoptosis after IR **(A)** (a–c) Apoptotic rates of the Control, NC and QSOX1-shRNA groups before IR. (d–f) Apoptotic rates of the three groups after 8 Gy IR. (**B)** Quantification of apoptotic rates before IR, both *p* > 0.05. (**C)** Quantification of apoptotic rates after IR. ^*^p < 0.05 vs. control; ^#^*p* < 0.05 vs. NC.

We further evaluated whether the increase in cell survival rate mediated by QSOX1-shRNA after IR is accompanied by a decrease in apoptotic cell death. Before IR, there was no dramatic difference in the rate of apoptosis among the control, NC and QSOX1-shRNA groups [(5.25 ± 0.28)%, (5.54 ± 0.45)% and (4.78 ± 0.34)%, respectively; all *p* > 0.05]. However, the apoptosis rates of the control and NC groups were significantly different from that of the QSOX1-shRNA group after 8 Gy IR for 24 h [(15.31 ± 1.39)% and (14.93 ± 0.64)% vs.(8.13 ± 0.64)%, respectively] (Figure 4). Therefore, to some extent, QSOX1-silencing exerts an inhibitory effect on the apoptosis of CNE-2 cells after IR.

### Results from the animal model

**Figure 5:**
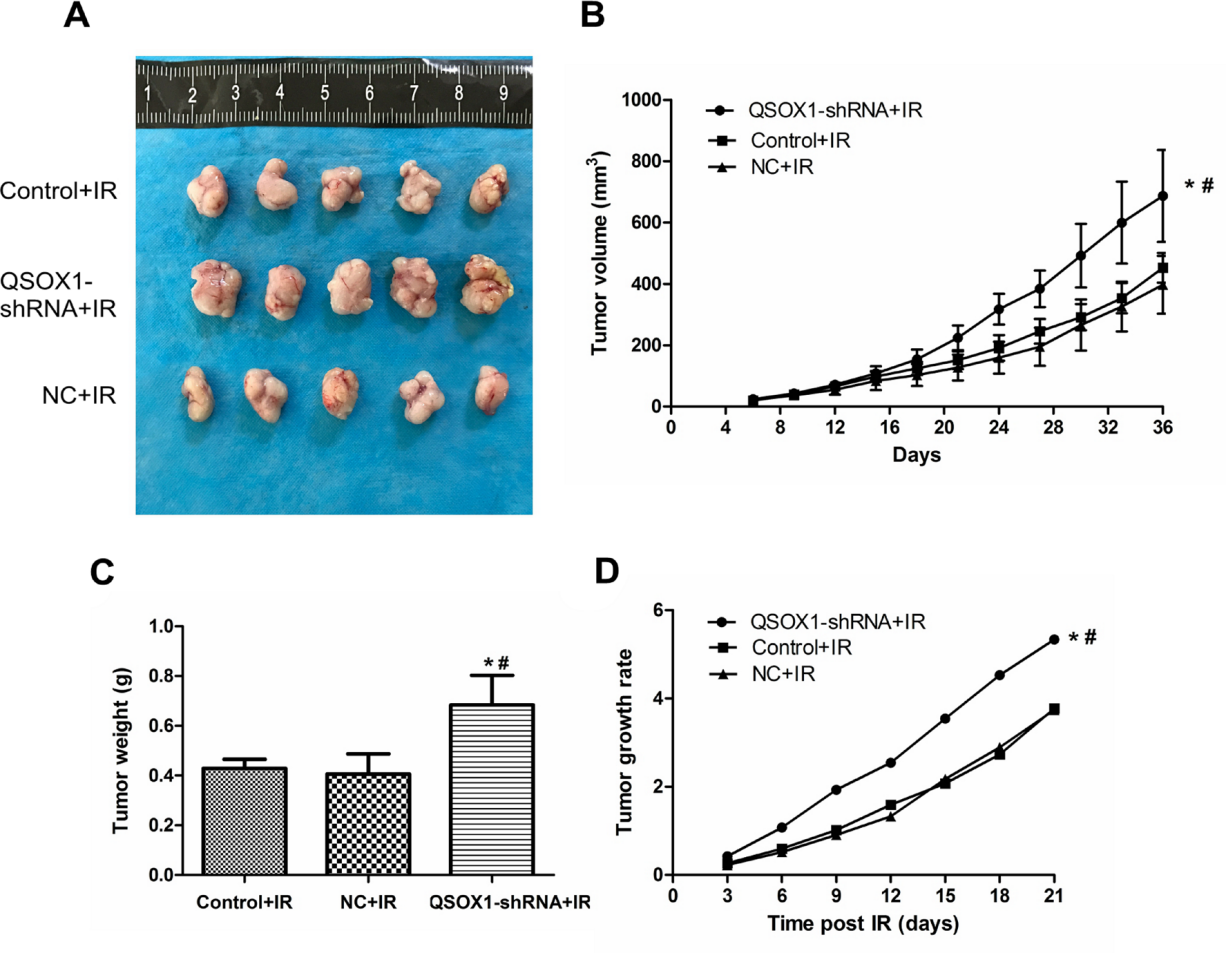
Knockdown of QSOX1 suppressed the radiosensitivity of CNE-2 cells *in vivo* **(A)** Representative images of xenografts excised from different groups exposed to radiation. (**B)** Tumor growth curves of different groups. Tumor volumes were measured every 3 days. (**C)** Tumor weights in the QSOX1-shRNA, Control and NC groups after IR. (**D)** Tumor growth rates. ^*^p < 0.05 vs. control; ^#^*p* < 0.05 vs. NC.

To confirm the QSOX1-mediated radiosensitization of CNE-2 cells *in vivo*, the three groups of cells described above were injected into nude mice. Mice continued to be fed for a period of 30 days after tumors were visible on the 5th day. There was no significant difference among the three groups before IR. However, the growth of tumors in the QSOX1-shRNA group was significantly greater than that in the control and NC groups (Figure 5A–5C) after 10 Gy IR. Furthermore, the rate of tumor growth was significantly higher in the QSOX1-shRNA group than in the other two groups (Figure 5D). Therefore, QSOX1-silencing weakened the antitumor effect of radiation and enhanced the radioresistance of CNE-2 cells inoculated into nude mice.

## DISCUSSION

Advancements in radiological techniques, Epstein-Barr (EB) virus screening and effective multimodal therapies have significantly improved the survival of NPC patients [1]. However, local recurrence, primarily caused by radioresistance, still limits the current clinical management of NPC [21, 22]. Therefore, prediction and prevention of radioresistance is imperative to further improve the therapeutic outcomes of NPC.

To identify biological markers that can predict the response of NPC to radiotherapy, in a previous study, we analyzed the profiles of secretory proteins in NPC cell lines with variable radiosensitivities using the quantitative iTRAQ method [8]. The resultant data identified 26 differentially secreted proteins, including QSOX1, that displayed a dramatic difference in expression between the radiosensitive and radioresistant NPC cells. In our current study, we detected the levels of QSOX1 in the sera and tissue samples of patients with NPC. To date, there are no effective biomarkers for predicting the radiosensitivity of NPC.

QSOX1 is an enzyme that oxidizes thiols to catalyze disulfide bond formation during protein folding and reduces oxygen to hydrogen peroxide. It is also a key enzyme involved in ECM modification by tumor cells. Therefore, QSOX1 participates in many cellular processes, including the DNA damage response, oxidative stress response and apoptosis induction [23]. The expression level of QSOX1 varies in different cancer cell lines [18, 19, 24]. In our study, according to western blot analyses, QSOX1 expression was weaker in the radioresistant CNE-2R cell line than in the parental radiosensitive CNE-2 cell line. Additionally, the detection of QSOX1 levels in serum and tissue samples of NPC patients using ELISA and IHC validated these results, and the radiosensitive patients exhibited higher QSOX1 expression (Figure 1). Therefore, we speculated that QSOX1 expression may be related to the radiosensitivity of NPC.

In this study, to determine the effect of QSOX1 on the radiosensitivity of CNE-2 cells, a QSOX1-silencing cellular model was established using a lentivirus-mediated shRNA silencing system (Figure 2A and 2B). Silencing of QSOX1 via shRNA weakened the response to radiation *in vitro* and significantly inhibited colony formation in CNE-2 cells (Figure 2C and 2D, Table 1). Similarly, the results from the CCK-8 assays indicated that the survival fraction of cells in the QSOX1-shRNA group after IR was dramatically higher than that of cells in the control and NC groups (Figure 2E), suggesting that silencing QSOX1 desensitizes CNE-2 cells to radiation.

Radiation has been shown to significantly contribute to the generation of reactive oxygen species (ROS) in a variety of cancer cells, which can effectively kill the cells [25, 26]. Low ROS levels is one of the major causes of radioresistance [27, 28], whereas elevated ROS levels lead to the induction of cell apoptosis and increase radiosensitivity [29–31]. Because the enzymatic activity of QSOX1 results in the production of hydrogen peroxide, the tumor microenvironment becomes highly oxidative, with elevated ROS levels [17]. In our studies, the radiosensitive group displayed a higher QSOX1 level than the radioresistant group, as demonstrated by proteomics analyses, western blotting, ELISA and IHC. Our data showed that after IR, the apoptosis rate of the QSOX1-silenced group was lower than that of the control group (Figure 4). Based on our results and the biological functions of the QSOX1 protein, we hypothesized that silencing QSOX1 may lead to a decrease in ROS production, which might contribute to the emergence of radioresistance in NPC cells. However, our results were not sufficiently convincing to support our hypothesis; therefore, additional validation should be performed.

The ability to migrate and invade through the basement membrane is a characteristic of metastatic cancer cells and modulation of the ECM by cancer cells may enhance tumorigenesis [32]. Cancer cells can alter the ECM by secreting or activating protein degradation enzymes, such as matrix metalloproteinases (MMPs), to breakdown matrix components [33]. Moreover, the cellular response to IR strongly depends on cell-ECM interactions [34, 35]. QSOX1 is a key enzyme involved in the ability of tumor cells to modify the ECM, and QSOX1 inhibition can undermine cell migration [16, 36]. Katchman BA *et al*. reported that QSOX1 plays a role in the growth and invasion of pancreatic and breast tumor cells *in vitro* [18, 19, 37], The authors also demonstrated that silencing QSOX1 prevents tumor cells from invading through Matrigel due to a reduction in the proteolytic activity of MMP-2 and MMP-9. Using a transwell assay, we evaluated the effect of QSOX1 silencing on the invasive ability of NPC cells. The shRNA-mediated knockdown of QSOX1 inhibited migration and invasion of CNE-2 cells (Figure 3). Furthermore, after exposure to X-rays, the invasion and metastasis abilities of QSOX1-silenced cells were significantly reduced compared with the control group. However, the underlying mechanism and whether this process is associated with the radiosensitivity of tumor cells remains unknown. Therefore, more focused studies are needed to address these aspects.

The role of QSOX1 in the response of NPC cells to IR *in vivo* was further investigated using a mouse model. Our findings suggested that QSOX1 knockdown increases the radioresistance of NPC cells *in vivo* (Figure 5).

Overall, our findings indicate that knockdown of QSOX1 in NPC cells enhances radioresistance both *in vitro* and *in vivo* and decreases cell apoptosis and invasiveness. In addition, QSOX1 expression in sera and tissue samples from radioresistant patients was lower than that in samples from radiosensitive patients. Thus, our findings indicate that evaluation of QSOX1 expression may enable the prediction of NPC radiosensitivity in patients and facilitate personalized treatment.

## MATERIALS AND METHODS

### Patients and samples

Serum and tissue samples were obtained from 60 NPC patients who had received radical radiation therapy at the Affiliated Tumor Hospital of Guangxi Medical University from January 2013 to May 2015. All of the serum samples were stored at −80°C, and the tissue specimens were fixed in 4% formalin and embedded in paraffin. Detailed clinicopathological parameters of the patients are listed in Supplementary Table 1. The samples were collected prior to radiotherapy. Patients enrolled were treated using a medical electron linear accelerator (Precise 1120; Elekta Instrument AB, Stockholm, Sweden) at a total dose of 68.2–72.32 Gy (2.18–2.26 Gy per fraction, with 5 daily fractions per week). Radioresistant patients were those who had residual lesions after 60 Gy of IR and experienced local recurrence within 2 years of treatment. Patients who achieved complete remission after IR with a dose ≤50 Gy and did not experience recurrence within 2 years of treatment were defined as radiosensitive.

### Determination of serum QSOX1 concentration via ELISA

The levels of QSOX1 in the serum samples of NPC patients were assayed using a human Quiescin Q6 Sulfhydryl Oxidase 1 (QSOX1) ELISA Kit (DL-QSOX1-Hu, Dldevelop, Canada) following the manufacturer’s instructions. The detection wavelength was set at 450 nm. Duplicate readings for each standardand samples were averaged after subtracting the optical density (OD) of the blank standard. Curve expert 1.30 software was used to construct a standard curve and calculate the QSOX1 concentration in each serum sample.

### Immunohistochemistry(IHC) for QSOX1expression quantitation

Briefly, after antigen retrieval, tissue sections were incubated with a rabbit polyclonal anti-QSOX1 antibody (1:200 dilution; Proteintech, Chicago, IL, USA) overnight at 4°C, followed by incubation with a biotinylated secondary antibody and an avidin-biotin peroxidase complex (ZSGB-Bio, Beijing, China). Then, the immune reactions were developed by adding DAB chromogen- substrate solution (ZSGB-Bio, Beijing, China) to the slides. Harris hematoxylin was used for counterstaining. Negative controls were run in parallel with all reactions. All specimens were scored by a board-certified pathologist. The scoring was determined as follows: i) the proportion of tumor cells with IHC staining for QSOX1 protein expression (0: no staining, 1 (Low): <33%, 2 (Intermediate): 33 to 66%, 3 (High): >66%), and ii) the intensity of the stain (0: negative, 1: weak, 2: moderate, 3: strong staining intensity). An overall staining score of ≤3 indicated low expression and a score of >3 was considered high expression.

### Lentivirus-mediated QSOX1 knockdown

To generate NPC cell lines with QSOX1 knockdown, shRNAs targeting three different portions of the QSOX1 gene (5′-TAGCCACAACAGGGTCAAT-3′; 5′CAAGAAGGTGAACTGGATT-3′ and 5′-CAATGTGGTGAGAAAGTTT-3′) were cloned into pLV-GV248-lentiviral vectors (GeneChem, Shanghai, China). An empty-vector lentiviral construct was used as the control.

### Cell culture and infection

The human NPC cell line CNE-2 used in this study was purchased from Fudan University Cancer Institute. Cells were cultured in RPMI-1640 medium (HyClone, Logan, UT, USA) supplemented with 10% fetal bovine serum (Gibco, Grand Island, NY, USA) and 1% penicillin/streptomycin (Beyotime Bio, China) (100 μg /mL), at 37°C in a humidified incubator with 5% CO_2_. The preparation of conditioned media (CM) was performed as previously described [8]. For lentiviral infection, CNE-2 cells were cultured with QSOX1-shRNA-encoded lentivirus (QSOX1-shRNA group) or empty vector-encoded lentivirus (NC group) at a multiplicity of infection (MOI) of 40 for 48 h. To assess the transduction efficiency, the expression of GFP was evaluated using an inverted fluorescence microscope (Olympus, Tokyo, Japan). Cell lines with stable knockdown of QSOX1 were selected using puromycin (0.5 mg/mL) for 2 weeks.

### Real-time RT-PCR

Total RNA was isolated from the cells with pre-cooled TRIzol reagent (Invitrogen, Carlsbad, CA, USA) and reverse transcribed with PrimeScript RT reagent Kit (Takara Bio, Otsu, Japan) according to the manufacturer’s instructions. The primer sequences for QSOX1 and GAPDH are summarized as follows: QSOX1 forward, 5′-GAGTTCTTCGCCTCCTGGT-3′ and reverse, 5′-TGTTGGTCTCCTCAGCACAG-3′. GAPDH forward, 5′-CACCATCTTCCAGGAGCGAG-3′ and reverse, 5′-TCACGCCACAGTTTCCCGGA-3′. GAPDH was used as the internal control. PCR was performed using SYBR Premix Ex Taq (Takara Bio, Otsu, Japan) and Light Cycler 480 software v1.5.0 (Roche Diagnostics, Switzerland). The QSOX1 expression was calculated by the equation 2^–ΔΔCt^.

### Western blot analysis

Protein samples were extracted from the harvested cells or conditioned media, and western blotting was performed according to standard methods. After lysis with RIPA buffer containing 1% protease inhibitors (PMSF), the CNE-2 and CNE-2R cells were harvested and centrifuged. Conditioned media (CM) of CNE-2 and CNE-2R cells were treated with a protease inhibitor tablet (Roche Applied Science, Mannheim, Germany) and concentrated to an approximate volume of 200 μL using a 10 kDa molecular-weight-cut-off Amicon Ultra-15 centrifugal filter device (Millipore, Bedford, MA, USA) following the manufacturer’s instructions. Proteins extracted from the harvested cells and conditioned media were electrophoretically separated on 10% SDS-PAGE gels before being blotted onto PVDF membranes (Thermo Scientific, Waltham, MA, USA). After being blocked with 5% non-fat milk in TBST, the membranes were incubated with a rabbit polyclonal antibody against QSOX1 (1:1,000 dilution, Abcam, USA) and a rabbit polyclonal antibody against GAPDH (1:1,500 dilution; Proteintech, Chicago, IL, USA) at 4°C overnight. GAPDH served as the loading control. Then, the membranes were incubated with a goat anti-rabbit secondary antibody (1:1,500) for 1 h at room temperature. Visualization was performed with an infrared fluorescence imaging system (Odyssey; Li-Cor Co., Lincoln, NE, USA).

### X-ray irradiation (radiation exposure)

X-ray IR was performed using a medical electron linear accelerator (Precise 1120; Elekta Instrument AB, Stockholm, Sweden), emitting at a fixed dose rate of 4 Gy/min.

### Cell irradiation and CCK-8 assay

CCK-8 assays were used to evaluate cellular viability in response to IR. Approximately 4000 cells/well were seeded in 96-well plates and allowed to adhere overnight. The cells were cultured for another 60 h after being irradiated with 6 MV X-ray at 2, 4, 6 and 8 Gy. Then, the cells were exposed to 10 μg/mL CCK-8 solution (Dojindo, Japan) for 1 h at 37°C. The corresponding OD values were then measured using a microplate reader (Bio-Rad, Hercules, CA, USA) at 450 nm. Each group was assayed in 5 duplicate wells, and three independent experiments were conducted.

### Colony formation assay

The radiosensitivity of cells was measured via colony formation assays following exposure to IR. Cells were treated with various doses of radiation after being seeded in 6-well plates at different densities (200, 200, 400, 600 or 1,000 cells/well with 0, 2, 4, 6 or 8 Gy of radiation). Then, the cells were incubated for another 12 days until colonies appeared. After being fixed with carbinol for 25 min and stained with Giemsa stain (Applichem, Germany) for 35 min, the colonies (each colony with at least 50 cells) were counted. Experiments were performed in triplicate. GraphPad Prism 5.0 software was used to fit the data to a multi-target, single-hit model; SF (survival fraction) = 1 – (1 – e^-*D*^/^*D*0^)^*N*^, where D_0_ (mean lethal dose) is the single dose of radiation that can kill 63% of the cells and N represents the number of intracellular radiation-sensitive areas.

### Cell migration and invasion assay

After being starved overnight, 1 × 10^5^ non- irradiated and irradiated (2 Gy) cells were suspended in 150 μL of serum-free medium and added to the upper portion of transwell chambers (Corning, USA), and 600 μL of complete medium was added to the lower chambers. For the invasion assay, five hours before cell seeding, the upper surface of the transwell chamber membranes were precoated with matrigel diluted in RPMI-1640 medium (1:8). After incubation for 24–32 h, the cells on the upper surface were wiped off with cotton buds. The remaining cells that had migrated or invaded to the bottom surface of the membrane were fixed with 4% paraformaldehyde and stained with Giemsa stain. The cells in five random fields from each membrane were counted under a microscope. The average cell number was calculated.

### Apoptosis analysis

Cell apoptosis was analyzed using flow cytometry. The cells were irradiated with X-rays at a dose of 8 Gy, collected after 24 h of culture and then incubated with 500 μL of 1 × binding buffer containing 5 μL of Annexin V APC and 5 μL of 7-AAD (BD Pharmingen, USA) for at least 15 min at room temperature in the dark. All samples were measured on an FC500 flow cytometry system within 1 h. Three independent experiments were performed.

### Xenograft mouse model

BALB/c nude mice (4 to 5 weeks old; SLAC Laboratory Animal, Shanghai) were used to establish xenografts by subcutaneous injection of 0.2 mL of cells (1 × 10^7^ cells/mL) into the right groin. The mice were then randomly divided into 3 groups. The tumor volume and activity of the mice were monitored and evaluated every 3 days. Tumor volumes were calculated using the equation, V (mm^3^) = 0.5 × a × b^2^ /2 (a is longest diameter, and b is shortest diameter). Radioresistance assays were performed when the transplanted tumor reached 80–120 mm^3^ (15 days). Mice were fixed on the boards in a supine position with their right hind leg stretched to expose the transplanted tumor. The tumors were irradiated with X-rays at a total dose of 10 Gy (5 Gy/week). In addition, the tumor site was covered with a tissue equivalent bolus. On day 14 after the last IR treatment, the animals were euthanized and the tumors were excised and measured. Tumor growth was calculated as follows: Growth rate = (V_t_–V_0_)/V_0_, where “V_t_” is the volume of each measurement and “V_0_” is the volume of the transplanted tumor the day before IR.

### Statistical analysis

Continuous data conforming to a normal distribution are presented as the mean ± standard deviation (SD) of at least 3 independent experiments, and the differences were analyzed using Student’s *t*-test or one-way analysis of variance (ANOVA). A Mann-Whitney *U* test was used to analyze the differences in serum QSOX1 expression level. All statistical analyses were performed with SPSS 17.0 (SPSS, Chicago, USA) or GraphPad Prism 5.0 software. Two-sided *p*-values < 0.05 were considered statistically significant.

## SUPPLEMENTARY MATERIALS TABLE


