# Supplementary material for: Reduced QSOX1 enhances radioresistance in nasopharyngeal carcinoma
Source: Oncotarget. 2017 Dec 14;9(3):3230–41. doi: 10.18632/oncotarget.23227 (PMC5790459; doi:10.18632/oncotarget.23227)
Supplement: Supplementary file 1 [file oncotarget-09-3230-s001.pdf]

Reduced QSOX1 enhances radioresistance in nasopharyngeal carcinoma

SUPPLEMENTARY MATERIALS

Supplementary Table 1: Comparison of clinicopathological parameters between the two groups of NPC patients

Classification		Radioresistant group (*n* = 28)	Radiosensitive group (*n* = 32)	$^{*}p$
Age	<50	17	20	>0.05
	≥50	11	12	
Gender	Male	22	25	>0.05
	Female	6	7	
Pathologic type	Differentiated	5	6	>0.05
	Undifferentiated	23	26	
T stage	T1–T2	12	9	>0.05
	T3–T4	16	23	
N stage	N0–N1	15	12	>0.05
	N2–N3	13	20	
Clinical stage	I–II	8	5	>0.05
	III–IV	20	27	
GTVnx (Gy)		71.18 ± 0.92	71.21 ± 1.01	>0.05
GTVnd (Gy)		68.33 ± 2.06	68.45 ± 2.42	>0.05
CTV1 (Gy)		60.50 ± 0.88	60.88 ± 1.01	>0.05
CTV2 (Gy)		54.45 ± 0.79	54.62 ± 0.87	>0.05

TNM staging was performed according with the 7th edition of the American Joint Committee on Cancer/Union for International Cancer Control (AJCC/ UICC) staging system. GTVnx = the gross tumor volume of nasopharynx, GTVnd = cervical lymph node tumor volume, CTV = clinical target volume. The differences between the two groups were not statistically significant.
